# Recent Advances in Bioprinting and Applications for Biosensing

**DOI:** 10.3390/bios4020111

**Published:** 2014-04-24

**Authors:** Andrew D. Dias, David M. Kingsley, David T. Corr

**Affiliations:** Department of Biomedical Engineering, Rensselaer Polytechnic Institute, 110 Eighth St., Troy, NY 12180, USA; E-Mails: diasa@rpi.edu (A.D.D.); kingsd@rpi.edu (D.M.K.)

**Keywords:** biofabrication, bioprinting, patterning, throughput, immobilization

## Abstract

Future biosensing applications will require high performance, including real-time monitoring of physiological events, incorporation of biosensors into feedback-based devices, detection of toxins, and advanced diagnostics. Such functionality will necessitate biosensors with increased sensitivity, specificity, and throughput, as well as the ability to simultaneously detect multiple analytes. While these demands have yet to be fully realized, recent advances in biofabrication may allow sensors to achieve the high spatial sensitivity required, and bring us closer to achieving devices with these capabilities. To this end, we review recent advances in biofabrication techniques that may enable cutting-edge biosensors. In particular, we focus on bioprinting techniques (e.g., microcontact printing, inkjet printing, and laser direct-write) that may prove pivotal to biosensor fabrication and scaling. Recent biosensors have employed these fabrication techniques with success, and further development may enable higher performance, including multiplexing multiple analytes or cell types within a single biosensor. We also review recent advances in 3D bioprinting, and explore their potential to create biosensors with live cells encapsulated in 3D microenvironments. Such advances in biofabrication will expand biosensor utility and availability, with impact realized in many interdisciplinary fields, as well as in the clinic.

## 1. Introduction

For diagnostic, environmental, and clinical applications of biosensors, it is necessary to be able to detect, quantify, and report a multitude of different analytes rapidly and simultaneously. Often, detection of a single analyte, such as a disease marker or protein, is not sufficient for diagnosis or disease screening. This has become especially relevant in recent years as patient-specific treatments are being developed based on patient-to-patient biomarker differences [1,2]. As the capabilities of biosensors continue to improve, these new biosensors must have the ability to multiplex large amounts of data, with high dynamic range and a low signal-to-noise ratio, ideally in a single sensor. Furthermore, as researchers develop a wide range of detection methods, the scaling and multiplexing of biosensing detectors and transducers becomes a biofabrication challenge. Current biosensors need to detect proteins, DNA, cells, and toxins, among other analytes. Generally, the detection of multiple analytes is preferred for performing diagnostics, monitoring, toxicity screening, and numerous other biosensing applications. 

These requirements demand sophisticated biosensor fabrication. Biosensors require multiple transduction elements in order to process multiple analytes separately, so high-throughput fabrication of transducers and/or detectors is of great benefit to the biosensor field. For example, recent biosensors have used high throughput protein patterning for bacteria detection [3], toxin detection [4], and measurement of protein interaction [5]. Additional types of high-throughput biosensor devices require sophisticated fabrication technology, such as micromechanical cantilever arrays to detect chemicals [6]. Alternatively, many recent biosensor-based studies aim only to detect single compounds, but strive to do so with very high sensitivity [7,8,9,10]. 

In order to detect multiple analytes or investigate biological outcomes with high throughput, it is necessary to harness biofabrication technologies for biosensors applications. Biofabrication technologies have been advancing in recent years, particularly cell-based biofabrication technologies that enable cellular encapsulation and growth in 3D microenvironments [11,12,13,14,15,16,17,18]. While there have been many recent biofabrication advances, particularly involving novel materials or combinations of materials [13,19,20], we will focus on those advances in printing and deposition technologies that may be most applicable to biosensors. 

It has been noted that miniaturizing sensors that depend on surface capture of molecules results in lower performance, due to transport of target molecules to the sensor [21]. However, patterning, as opposed to flow-based delivery, allows direct delivery of molecules, cells, or a sample to be analyzed to a desired location. This direct delivery substantially improves binding kinetics over random interactions. Pattern-based high-throughput biosensing typically involves depositing a protein, antibody, or other molecule of interest to a substrate, and measuring binding of another molecule or cell [5,22,23,24]. Patterning is typically achieved using soft lithography-based techniques, which use elastomeric stamps with small relief features fabricated from silicon wafer masters [25,26]. For lithography-based patterning applications, a photomask is used to enable selective polymerization of photoresist, on silicon, to create a master. In the example of microcontact printing, this master can then be used as a negative to fabricate multiple elastomeric stamps or molds, typically out of polydimethylsiloxane (PDMS). Self-assembled monolayers (SAMs) of various polymers can be patterned onto surfaces using a stamp, although chemical interaction between the patterned polymer and the substrate limits the polymers and substrates that can be used. Soft lithography techniques are contact-based, and involve high pressure on a stamp to transfer material from a donor substrate. Alternatively, patterning can be achieved using non-contact deposition methods, including inkjet printing and laser direct writing (LDW). Non-contact methods allow the direct patterning of material, or even cells, where the specific binding chemistry is not as critical. 

Non-contact deposition methods, such as LDW, hold distinct advantages over other techniques for biosensor applications. Typical contact-based patterning involves a chemical or physical interaction with the substrate, and usually requires restricting growth to a specific domain. The unrestricted growth of cells is a necessary criterion for routine cell function. With uncharacteristic cell function, cell analyte production may be affected, thereby propagating an error in the biosensing system. To circumvent substrate modification that may influence cell function, non-contact based methods are employed. LDW and inkjet printing are both non-contact bioprinting methods capable of high-throughput non-contact deposition. These two systems differ in their performance, with distinct trade-offs in throughput and accuracy/precision. LDW is a more accurate and precise deposition technique, while inkjet printing offers greater throughput.

For high-throughput applications, patterning methods typically immobilize proteins via adsorption to a surface. However, many biosensors are live-cell devices that sense biological functions and cell behavior in response to stimuli. Simply measuring the response of a cell to a static factor may not be sufficient for some applications, and information about dynamic response and longer-term changes in cell behavior may be desired. While there are some biosensors that can monitor certain molecular levels, such as real-time glucose monitoring *in vivo* [27], real-time measurement systems generally do not extend beyond one analyte because of the specific detection methods [28]. Some technologies that examine real-time changes in cell behavior include Förster resonance energy transfer (FRET), bioluminescent resonance energy transfer (BRET), or other fluorescent-based detection systems that respond to a binding event [29]. However, these techniques are also typically limited to a few analytes because of fluorescence emission overlap. Overall, the throughput achievable in real-time biosensing is somewhat limited.

Biosensing may benefit from the application of patterning technologies to produce live cell devices, because patterning allows the placement of specific analytes or cells in defined spatial locations. Patterning separate analytes, or even transducers, in parallel, defined locations can be used to test specific stimuli simultaneously. This simultaneous testing not only increases throughput, but does so in a manner that synergistically increases the information one can glean from analytes and cellular responses. Herein, we examine various patterning technologies, including those that can pattern proteins and viable cells. Recent biofabrication advances in patterning, particularly with live cells and in 3D, may help to advance the biosensors field. It is becoming clear that high throughput and rapid screening of multiple markers may be necessary for point-of-care diagnostics. However, examining dynamic cellular response via live-cell assays is also an important feature that needs to be addressed by new biosensors, and this may be feasible using high-throughput methods.

## 2. Transduction and Detection Methods for Biosensing

**Figure 1:**
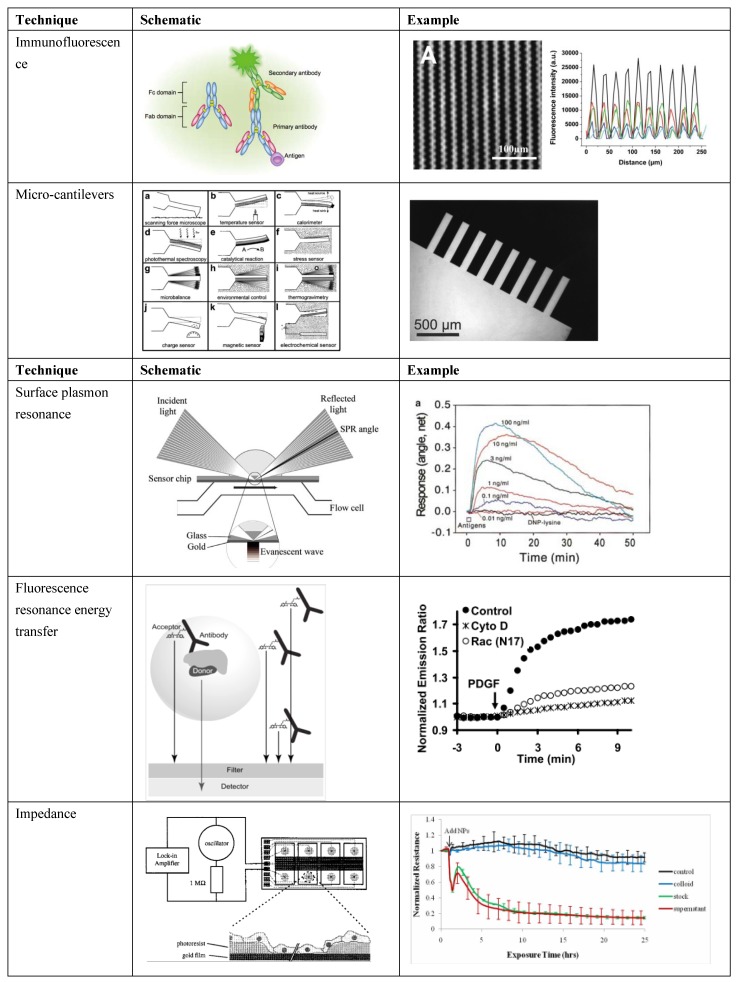
Select biosensor transducer schematics and examples of use. Immunofluorescence schematic [30] and example involving sensing in microgrooves [24]; microcantilevers schematic [6] and example of a fabricated microcantilever array [6]; SPR schematic [22] and example involving detection of concentration of a molecule over time [31]; FRET schematic [32] and example [33] of signal in perturbed and normal cells; impedance schematic [34] and example of signal in control and cells with a toxin [35] (Adapted by permission from Macmillan Publishers Ltd.: *J. Invest. Dermatol*. [30], ©2013; reused with permission from Elsevier [6,22,24,31,32,34]; with permission ©2008 by the National Academy of Sciences [33]; with permission from Inderscience Ltd. [35], ©Inderscience 2011, respectively).

Biosensors, even those fabricated with sophisticated bioprinting techniques, consist of three elements: the biological signal (analyte), the transducer(s), and the detector/readout. The biological signal is what one is trying to detect, while transducers permute this signal into one that can be detected and measured for analysis. The biological signal occurs naturally, so most of the engineering involves the transducer and detector. Furthermore, detectors primarily can report changes in light, voltage, pH, and/or absorbance, so more engineering freedom lies in the transduction elements than in the detector. In order to detect analytes at small scales, sophisticated transducers must be designed that can identify, quantify, and potentially localize a material, antibody, chemical, or other biologic. To detect a small amount of signal, an event (e.g., chemical binding, a micromechanical response, or a change in cell behavior) must be transduced and amplified such that it can be quantified, displayed, and compared to desired values. Transducers, therefore, must be a robust part of the biosensor, because they are the only element that actively transforms a local event to a measurable signal. Depending on the application, a variety of transducers can be employed, and many common transducers are shown in Figure 1. While this review will focus on patterning and bioprinting approaches useful in biosensing, a transducer must be carefully chosen, depending on the biosensing application. Therefore, we present transducers that may be selected when designing a biosensor. Each offers unique benefits and challenges, with some transducers better suited for applications in multiplexing, real-time biosensing, or fabrication.

One common transduction method is specific ligand binding [36]. When a ligand of interest binds to a cell [31,37], antibody, protein, or chemically-modified surface [38], the binding event can be detected by various methods. Methods for detecting fluorescent protein-protein binding include enzyme-linked immunosorbent assays (ELISAs) [5], which can be performed in high-throughput fashion. However, these data represent a static biological configuration, (*i.e.*, cells are typically lysed), so any temporal information of the response is lost. Other static biosensors include the patterning of protein, or a chemically active transducer onto a substrate to detect an analyte [24,39,40]. The optical absorbance of a system is often used to measure protein concentration, and when fluorescent labeling methods are used, multiple analytes can be detected simultaneously. Ligand binding events can also be detected on a macro scale via secondary transducers, such as a change in absorbance, surface plasmons, voltage, or other properties. These methods enable dynamic biosensing because they allow cell survival during measurement, or even *in vivo* measurement, and any changes in the system are reflected in real-time. Amperometric sensors, for example, have been used to report real-time glucose concentrations [41] and to detect micro RNA [42]. 

Recently, more sophisticated and sensitive transducers have been employed for real-time biosensing. Surface plasmon resonance (SPR)-based transducers, which measure the resonance angle of reflected light from a gold-coated surface in response to a specific binding event to immobilized receptors, have been used to detect cell/ligand binding and release [37] or specific molecules [22]. SPR takes advantage of charge density oscillations at the interface of two materials with opposite dielectric constants [43], and by careful choice of the optical system and the transducing medium to the analyte, this technique can potentially be very sensitive. Related techniques, such as localized SPR, where nanostructures or nanoparticles are introduced to the system, may provide even greater sensitivity [44]. 

Detection systems like SPR permit cell survival over a long-term experiment, which allows measurement of a cellular response to transient inputs [22]. Similarly, resonant waveguide biosensors, which incorporate a diffraction grating [45,46] have been used to measure longer-term cellular responses, such as receptor sensitization and cellular response to stimuli [47]. SPR has been used to multiplex the detection of multiple molecules [48], but because there is only one output channel, simultaneous detection and quantification of multiple analytes is difficult. Miniaturizing a multiplexed plasmonic sensor has been accomplished using computational imaging at resonant wavelengths to detect resonance shifts [49]. 

Specific binding may also change surface stress and actuate a device like a microcantilever [6,50,51,52,53] for detection. A mechanical displacement of the cantilever due to a specific binding event can be detected by a piezoelectric sensor, or laser deflection on a photodetector. Cantilevers arranged in parallel systems have been used to detect multiple analytes by placing a distinct antibody or chemical on each cantilever. This high throughput use of cantilevers has been billed as an “artificial nose” [6]. Microfluidics have also been used to scale ligand binding approaches [54,55]. Microfluidics can be used to flow proteins, specific antibodies, or analytes to specific locations. After molecules flow over and bind to the substrate, they can be used to capture analytes and bind fluorescent antibodies. Both microcantilevers and microfluidics allow the location of binding to be controlled. Location control enables multiplexing many components onto a biosensor, because each component is separate, and can be detected independently.

However, binding is not always necessary for transduction and detection, and ligand binding may be used in tandem with other transduction methods. In biological systems, a small change in the microenvironment can cause a measurable change in pH or absorbance, signal cells to produce a factor that can be detected, or change the complex impedance of a membrane. pH changes and absorbance changes are not always biologically relevant, particularly in real-time systems. Healthy cells require a defined pH, and measuring absorbance is most useful after introducing fluorescent labels to the system. Sensitive approaches for real-time biosensing that do not require a binding event often require a sophisticated transduction system. Other detection methods for system-wide changes in cell behavior include impedance-based transduction, such as Electric Cell-substrate Impedance Sensing (ECIS), in which a membrane impedance is measured, and impedance changes due to cell behavior can be detected, such as those experienced in response to the presence of a toxin [34,35,56,57,58]. ECIS has been miniaturized to a biochip [4], thus ECIS may be scalable if many such chips can be fabricated. SPR signals have also been used to transduce a cellular response to a stimulus [31]. System-wide changes, like ECIS and SPR, may be useful for the detection of single analytes, but when multiplexing detectors or when multiple analytes are necessary, more precise fabrication methods may be required for increasingly complex sensors. However, sensors that can detect changes in impedance or SPR across a membrane allow the detection of reversible responses on cells, and a return to system equilibrium after a stimulus is removed. It is desirable to detect changes in cellular behavior over time, as cells may recover from a stimulus or have a delayed response. While these methods allow cell survival and can be used for real-time biosensing, the main disadvantages of transduction methods that do not require ligand binding are the limited throughput and lack of specificity to what is being detected.

Current biosensor applications can utilize cells in a variety of ways to produce cell-based biosensors. In these technologies, cells can function in various roles for the detection system. Previously, we describe how ligand binding can be applied directly to cells with the use of enzyme-linked fluorescence. In this application, the transducing element is the fluorescent antibody bound to a specific protein. However, there are several other applications of cell-based biosensors that utilize live cells as the transducing element. Cells possess natural mechanisms to transduce specific physiological analytes or environmental changes, thereby offering a unique advantage for use as transducing elements. There are many current examples of cell-based transducers in biosensing. One such example is the use of artificial neural networks on an electrode, where the neurons convert an environmental change to electrochemical signal [59]. Here, neuroblastoma cells were sensitive to specific compounds in pesticides, but similar biosensors could be designed with cells sensitive to different compounds. In another example, environmental changes cause differences in cell metabolic activity that can be measured by changes in the extracellular pH [60]. Here, the rate at which cells excrete acidic products of metabolism was measured in the presence of different materials. Moreover, a cell can transduce the signal from an analyte or environmental factor interacting with cellular receptors to a specific protein or soluble factor via secondary cellular messengers and a physiological response. This soluble factor or protein can then be directly detected using methods such as fluorescently tagged antibodies, or by binding to a secondary cell that will produce a response to the environmental change detected by the primary cell. This type of signaling occurs in blood sugar homeostasis, where pancreatic beta cells in the presence of high glucose concentration secrete insulin that will signal secondary cells to convert glucose to glycogen. 

Especially at small scales, it is important that biosensing capabilities have high sensitivity and specificity, as well as rapid response times. For biochemical detection by specific binding, these requirements are met easily, but the challenge in developing the capability lies in choosing and building the specific binding factor of interest. However, when biosensing involves changes to cell behavior, or a long-term change in the system, responses may occur at much longer time scales. An ideal biosensor is thus one that is specific, can record reversible responses, operates in real-time, and can allow high-throughput detection of multiple analytes. While the biosensor transducers mentioned above may meet some of these requirements (Table 1), new fabrication techniques may enable the next generation of transducers to meet many more. In particular, bioprinting approaches can be used to deposit specific proteins or cells in desired locations, which allows for high throughput and multiple analytes. The versatility of biofabrication and bioprinting will enable robust biosensors to be developed.

**Table 1:** Selected transduction methods and rated performance measurements for biosensing.

Transduction method	Transduction mechanism	Simultaneous multiple analyte detection +++	Real-time capability +++	Fabrication speed and customizability +++
Immunofluorescence	Fluorescent molecule specific binding	++	+	+++
Micro-cantilevers	Actuation during binding event	+++	+	+
Surface plasmon resonance	Adsorption or binding changes local index of refraction and resonance of surface plasmon waves	-	+++	+++
Resonance energy transfer	Specific binding or interaction enables emission from and detection of target	++	+++	+
Impedance	Impedance of cell membrane measured	-	+++	++

## 3. Methods for Bioprinting and Applications to Biosensing

Bioprinting involves the transfer of material and/or cells to a substrate. It offers many capabilities that can be utilized in biosensing applications, including rapid deposition and patterning of proteins or other biomolecules. There are many bioprinting technologies that can be adapted for use in fabricating biosensors. Some printing techniques, such as electrodeposition, may enable the transfer of thin films of metal nanoparticles [61] or nanowires [62] to a substrate, via an electric field. Printing thin films of metals can be utilized to create circuits that may be an integral part of a biosensor, as well as for some immunoassays or microarrays [63,64]. Electrodeposition has even been applied for printing thin films of biological material such as proteins, enzymes, nucleic acids, polysaccharides and bacterial cells [65,66,67]. While thin films have been used for biosensing applications, we will focus on bioprinting techniques that can be used to deposit a large range of biologics and mammalian cells in precise spatial locations. Directly printing biologics and cells enables use of the transducers described in Section 2. Some examples of these bioprinting techniques are shown in Table 2. Moreover, spatial precision can enable multiplexing and high-throughput analysis, to rapidly screen and detect multiple signals. For instance, rapidly patterning multiple proteins at different concentrations can enable detection of threshold levels to elicit a cellular response, or to promote cellular adhesion for parallel experiments. This review does not include details on techniques used to create a thin film without spatial control, such as electrodeposition. However, electrodeposition can be combined with stamping or masking [68,69] (which we review in this paper), or with a microarray or pattern of electrodes [70,71], to create a thin film with spatial control.

**Table 2:** Selected patterning biofabrication methods, performance, and some materials that have been patterned.

**Patterning Technique**	**Technique mechanism**	**Resolution** +++	**Throughput** +++	**Printable materials library** +++
Microcontact printing	Contact-based method, typically microstamp.	+++	++	++
Ink-jet printing	Droplet ejection from nozzle invovling thermal piezoelectric or pressure	+	+++	++
Matrix-assisted pulsed laser evaporation direct-write	Non-contact deposition via pulsed laser directly onto gel with cell suspension	+++	+	+++
Laser-induced forward transfer	Non-contact deposition via pulsed laser on sacrificial layer	+++	++	+++

**Figure 2:**
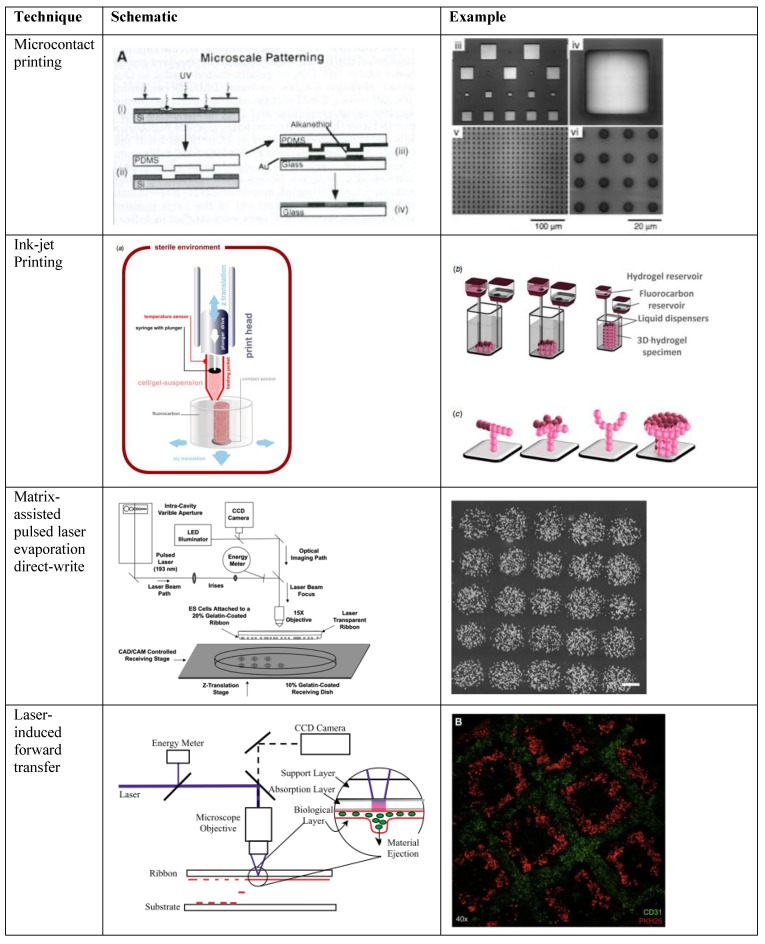
Schematics and examples of selected biofabrication patterning techniques, including microcontact printing [72], inkjet printing [73], MAPLE-DW [74], and LIFT [75,76] (Used with permission from John Wiley and Sons [72]; ©2013 IOP Publishing, reproduced with permission, all rights reserved [73]; used with permission from Elsevier [63,74,75,76], respectively).

In order to multiplex either the detection of different analytes or the effect on different types of cells, components of the system must be spatially separated. With many transduction techniques, different analytes can interfere with each other. Moreover, if various concentrations of a single analyte need to be tested, multiplexing by spatial separation of experimental conditions is required. Printing cells at various colony sizes, or proteins at different concentrations, may have an effect on cellular behavior, and can influence how cells respond to exogenous signals. Numerous printing approaches are available, and they can generally be divided into two major categories: contact-based and non-contact-based printing. Contact-based approaches require direct contact between a receiving substrate and the surface from which a material is donated, often with high pressure, to cause transfer to the receiving substrate. Non-contact-based printing, on the other hand, involves the transfer of material or cells to a receiving substrate via an ejection event. Upon ejection, the transferred material is neither touching the donor nor receiving substrate. Both printing approaches have merits and shortcomings, depending on the desired application. A variety of printing approaches is illustrated in Figure 2.

### 3.1. Contact-Based Bioprinting

Contact-based printing methods involve a mask or material that touches the substrate to constrain the cells or biological material to a specific area. Many contact-based printing techniques are based on soft photolithography. Precise micropatterning of proteins can be accomplished by using a photolithographic mask and photoresist, combined with chemical treatments to directly create protein micropatterns from a silicon master [77], or to fabricate stencils [78] or microstamps [72] from polydimethylsiloxane (PDMS). The micropatterns are created when the master or stamp is coated with the desired material, and stamped with high pressure onto the substrate. In addition to their use in contact deposition from a master or mold, removable masks have also been fabricated to shield regions of the substrate [79,80]. When this sort of mask is used, the stencil is applied to the substrate, washed with protein or cells, and then removed. Cells or proteins are only deposited on the unshielded regions. After the mask is removed, a high-fidelity pattern is obtained. Using these lithography-based techniques, proteins can be precisely patterned on flat, or even curved [81] surfaces, which is useful for controlling cell adhesion or other responses. Microcontact printing, in particular, can be used to deposit multiple layers of proteins, or to fabricate hollow microfluidic channels, by creating a stamp with three-dimensional features [82]. Microfluidic channels, in turn, can also be customized to allow valves and pumps [83,84,85], both of which are useful in biosensing applications because of the potential to control input of an analyte. Such microfluidic systems enabled by lithography-based technologies allow delivery or removal of fluids to specific areas, and creation of gradients [86] of soluble factors that can signal cell behavior. Three-dimensional fluid delivery [87] grants the ability to fabricate highly complex patterns and to deliver proteins, drugs, or cells to discrete regions of a microenvironment. These sensing devices [88] combine the high-throughput aspects of microfluidics with cell imaging to acquire large quantities of data in real-time.

Contact-based printing is particularly useful for transferring a specific protein to a surface with micron-level resolution. Protein patterning can be accomplished by direct transfer from a master or reusable stamp, using a removable mask to prevent cells from attaching to undesired regions of the substrate, or indirectly, by fabricating microfluidic channels. Channels fabricated by contact-based printing can have similar patterning resolution as direct patterning, but they also allow non-contact deposition with the ability to flow fluid to controlled locations. Directly printing proteins has been used to control cell shape [72,89,90,91], migration cues [92], and to analyze stem cell differentiation [93,94]. Microfluidic chambers have been utilized in genetic analysis and sequence identification [88,95] single-cell lysis [96], detection of circulating tumor cells [97,98], cellular differentiation [99], and delivery for *in vitro* drug testing [100]. Although they offer high spatial precision, lithography-based patterning techniques require the fabrication of a new stamp if a different pattern is desired, and generally cannot directly pattern cells to a surface or transducer because of high pressures or temperatures involved in the process. Repeated micropatterning on the same surface may also be difficult because the stamp and substrate must be precisely aligned [90]. While the aforementioned techniques have applied protein patterning to a hard substrate, recent micropatterning has also crosslinked hydrogels through a photomask [101], enabling 3D cell culture. 

In addition to these lithography-based methods, contact-based printing methods also include atomic force microscopy (AFM)-based deposition (reviewed [102]) such as dip-pen nanolithography (DPN) [103]. DPN uses an AFM tip to deposit an “ink” onto a solid surface with an affinity for the ink, analogous to microcontact printing. Multiple materials have been demonstrated for this technique [104], but the ink and substrate must still have an affinity for one another. AFM-based methods offer sub-micron level resolution, and even nanometer-level printing resolution. As cells can sense scale at the nano level [91,105,106], this sort of patterning may prove useful, particularly with sensors involving cells. Overall, contact printing and microfluidics can be applied to a variety of cell-based and protein-based biosensors at multiple scales.

### 3.2. Non-Contact Printing

Non-contact printing techniques involve transfer of a material to a substrate, with only the transferred material coming into actual contact with the substrate. Therefore, the high pressures associated with a stamp, or removal of a mask from the surface, are not required, making this approach more appropriate for softer materials. Non-contact printing offers a greater versatility in the substrates onto which materials can be printed, such as soft hydrogels, and the non-contacting nature allows layer-by-layer deposition to be utilized. There are various non-contacting methods to deposit biological materials, including viable cells, to substrates in controlled patterns. Non-contact printing is less reliant on surface chemistries than contact-based approaches, allowing deposition of a wider range of materials, to a wider variety of receiving substrates. However, along with this gained versatility, there can be a loss in printing resolution compared to contact-based approaches. For biosensing applications, biomaterials and bio-inks are critical when transducing a biological signal. Non-contact printing methods offer a wide range of material options, which, in turn, provide greater biosensor customizability. While additive fabrication techniques may be compatible with a wide range of materials, only a subset can be used for biosensing, because the material may have to be biocompatible, have a specific affinity, or fall within a specific viscosity range. From a fabrication standpoint, viscosity is particularly important in nozzle-based printing approaches because of potential clogging issues [42]. From a biological perspective, cells can also sense and respond to materials of different viscosities and stiffnesses [107,108]. Other characteristics of materials that may influence biosensing include the hydrophobicity/hydrophilicity of the substrate, as well as the pore size in the case of 3D geometries. These properties can affect ligand and cellular binding, as well as cellular behavior. 

A specific non-contact printing approach may be selected based on the biosensing application and desired material properties within the system. A common technique for printing relatively non-viscous materials is inkjet printing, which usually involves thermal [109], piezoelectric [110], or pneumatic [111] ejection of a droplet from a nozzle or printhead [112]. Inkjet printing has been adapted to print both proteins and live cells [109,110,113,114]. Conventional inkjet printers have been adapted for biomaterials and cells [115], and layer-by-layer printing allows for the fabrication of complex structures. While high throughput is possible, and pattern customization, including multiple cell or biomolecule types, is relatively easy, inkjet methods have lower resolution than lithography-based and laser-based patterning methods [114,116,117,118]. The high-throughput aspects of inkjet technology are appealing to biosensor applications, but defining locations for precise ligand binding or specific molecular interfaces may be difficult.

Various laser-based approaches have also been used for non-contact material patterning. Lasers are often used to directly crosslink photo-active materials into desired patterns using a photomask [119,120] or two-photon polymerization [121], but, herein, we will focus on methods used for direct deposition of a material. In addition to the ability to transfer soft materials, laser-based deposition methods allow for a wide range of materials to be used, far beyond those that can be laser crosslinked. This makes laser-based deposition particularly attractive for biosensing applications. LDW is a forward transfer technique used for printing viable cells, proteins, soluble factors, microbeads, and DNA [122,123,124]. A typical LDW setup consists of two coplanar plates, a “print ribbon” and a receiving substrate. The ribbon contains material to be deposited to the substrate, with an underlying coating consisting of two parts: a sacrificial layer and a transfer layer. The laser is focused on the sacrificial layer, and when the laser is pulsed, the sacrificial layer desorbs and ejects material from the print ribbon onto the receiving substrate, which can be moved to fabricate a programmed array or structure of biomaterial or cells. Often, the receiving substrate is coated with a hydrogel to cushion the ejected material. There are currently several different variations of LDW specifically modified for bioprinting, yet, the underlying principles of material deposition remain unaltered.

Matrix-assisted pulsed-laser evaporation direct-write (MAPLE-DW) is a commonly used form of LDW [125,126]. The unique aspect in the technique of this variation of LDW is that a single matrix is used as the sacrificial layer and the transfer layer. Typically, the ribbon coating is a polymer or hydrogel, such as Matrigel [127] or gelatin [122], that suspends the desired transfer material [122,126]. MAPLE-DW has previously been shown capable of fabricating simple biosensors, such as a dopamine electrochemical sensor [128]. 

Laser induced forward transfer (LIFT) is another commonly used form of LDW [129]. LIFT utilizes a ribbon with a distinct sacrificial and transfer layer. The sacrificial layer in a LIFT setup is typically a thin metal film that can be volatilized by the laser used. Below the metal film is a layer with the transfer material, typically some sort of polymer or hydrogel. Like MAPLE-DW, LIFT researchers have experimented with fabrication of biosensors. As a demonstration of LIFT’s biosensor fabrication ability, a DNA microarray was created with specific DNA strands, which could be detected by fluorescence microscopy when hybridized with complementary fluorescent strands [130]. Conventional equipment can be used to detect printed material and/or interacting analytes, and the pattern resolution afforded by LIFT allows customization and rapid fabrication of a biosensor. While LIFT and MAPLE-DW follow the same mechanistic principles, LIFT may offer additional shielding of the transfer material because of the specific sacrificial layer. 

LDW has many distinctive differences from other biological printing and patterning techniques. First, LDW has demonstrated its capability or potential to print with a wide range of polymeric material, which is essential for biological materials, especially viable cells. Some of the materials currently used include alginate, gelatin, Matrigel, collagen, fibrinogen, chitosan, and hyaluronic acid [12,125,131]. This wide range of possible hydrogels or hydrogel blends gives users the ability to pick specific materials for their desired outcomes. For example, when minimal interaction between cells and the biomaterial is desired, gelatin may be chosen because its crosslinking is thermally reversible, freeing cells to migrate unhindered. Further, the ribbon can be quickly changed during LDW, allowing the user to change transfer material, as is necessary for creating a co-culture cell system. Additionally, LDW allows deposition to any planar receiving substrate material. For patterning involving cells, there is traditionally a thin film used for cushioning the impact of the ejected material. A variety of different hydrogels have been used as a cushioning layer or as a post-printing scaffold. New methods have even used transplantable surfaces such as cardiac patches that are transferred to *in vivo* environments after printing [76]. Other traditional patterning and printing methods can be limited by factors including material viscosity and high shear rates, thereby greatly restricting their candidate material choices.

Throughput, accuracy, precision, and customizability are important features to consider in technologies used for fabricating biosensors. LDW has demonstrated the ability to create patterns of customizable spacing with 5 um resolution [122]. The large degree of spatial control of the deposited material enables the fabrication of intricate and highly sensitive biosensors. Additionally, LDW is a fully automated system with CAD/CAM controls. This control allows for not only fully automated rapid fabrication of complex designs, but for additional customizability of patterns that can vary between receiving substrates. However, LDW’s high degree of spatial control over deposited material can make large-scale fabrication impractical because of the time required to generate a large, high-resolution pattern. In some cases, it may be more appropriate to use an alternate patterning approach, or a combination of approaches.

## 4. *In Situ* Crosslinking for 3D Bioprinting

Two-dimensional cell culture has been the platform for many important scientific discoveries, ranging from studies involving stem cell differentiation to cancer drug screening. However, there are limitations on the translatability of 2D cellular system models, because there are major differences between the way cells behave and function in 2D and 3D environments [132], particularly because of cell adhesion and adhesion-based signaling [133]. When examining malignant breast cancer cells, it has been demonstrated that the 3D adhesion pathways are coupled, yet they are independent in a 2D model [134]. In the scenario of drug screening, the response of 2D and 3D surrogate cultured cells may profoundly differ due to this type of functional difference. For example, when incorporated into a biosensor, cells cultured in 2D likely will have different functional responses to varying conditions because of the difference in cell-matrix interactions from 2D to 3D culture. To more closely model the *in vivo* cell environment, many researchers have transitioned to 3D cell culture platforms using various materials to represent the cell environment.

Many different materials are currently being used to create 3D environments for cell studies. With the goal of mimicking the natural tissue environment for the cell type cultured, various material factors must be considered, such as elastic modulus, hydrophobicity, matrix interaction points (e.g., cell binding domains), material degradation rates, and porosity [132]. To control these properties, naturally occurring polymers found in the body are often used to engineer 3D cell scaffolds. Some naturally occurring polymers currently used in 3D cell culture include collagen, hyaluronic acid, and fibrinogen/fibrin. Additional polymers, not naturally occurring in the human body, such as alginate and chitosan, are being utilized for their unique and desirable characteristics. 

Small-delivery vehicles, namely microbeads and microcapsules, are being investigated for applications within biosensor technology. Microbeads are micron-scaled spheroids, made from crosslinkable polymers that are used in hydrogel fabrication. Currently, these 3D microenvironments are being explored for purposes involving sequestration of small molecules, proteins, drugs, or encapsulation of cells [135,136,137]. As microbeads are made from hydrogel material, they are approximately 98% water by mass, allowing for rapid diffusion into and out of the bead. Additionally, by adjusting properties and/or processing of the hydrogel polymer, many features of the microbead can be customized, such as pore size, elastic modulus, degradation rate, and permeability. These features allow for unique biosensor fabrication utilizing microbeads with encapsulated molecules or cells. As the biological element in the microbead is consumed or released, the transducer will indicate a biological response. 

In order for a biosensor to function properly, it may require the immobilization of a biological element on a transducer. However, living cells are not easily immobilized, and may migrate away from the transducer on a homogenous substrate. Similar issues arise when trying to localize cells into a particular area while they are proliferating and experiencing 2D phenomena. Cell encapsulation is one possible solution to this problem. Microbeads and microcapsules are popular micro-encapsulation technologies, at a scale appropriate for use in a biosensor. Similar to microbeads, microcapsules are micron-scale spheroids, however, rather than a 3D matrix, a spherical outer shell constrains the geometry, within which encapsulated cells may migrate. Further, these technologies offer the ability to tailor the size of the environment and total number of encapsulated cells. Traditional microbead and microcapsule fabrication technologies are unable to precisely place the fabricated structures in specific locations, which would be necessary to transduce multiple signals. However, a new method allowing for the one-step fabrication and patterning of cell-containing microbeads could solve this problem [124].

**Figure 3:**
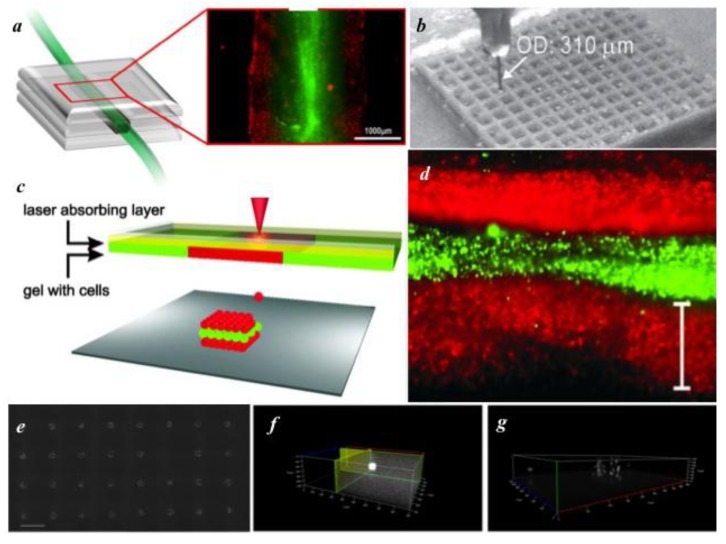
Examples of 3D patterned structures with future biosensing application. (**a**) inkjet printed vascular graft [145], (**b**) continuous flow printed structure [146], (**c**) laser patterned cell arrays in hydrogels [144], (**d**) skin graft fabricated with alternating cell layers [144], (**e**) laser patterned cell containing microbeads [124], (**f**) structure of 3D microbead based on an z-stack image of a rhodamine-containing microbead [124], and (**g**) z-stack image of 3D distribution of cells within an microbead [124] (Used with permission from Elsevier [145]; used with permission from John Wiley and Sons [146]; ©2013 IOP Publishing, reproduced with permission, all rights reserved [124], respectively).

For the fabrication of 3D constructs, there are several different routes researchers are pursuing; examples of such constructs are shown in Figure 3. One approach utilizes injection molding to fabricate large constructs [138]. In this procedure, cells are suspended in a crosslinkable hydrogel, or hydrogel blend, before injection into a mold and subsequent cross-linking of the hydrogel [138,139]. This technique allows for the fabrication of large or small constructs, with the overall geometric control being limited only by the quality/detail of the mold. New image-guided technology utilizing MRI and micro-CT allow for the fabrication of highly detailed personalized molds [138]. In theory, injection molding is compatible with any crosslinkable polymer solution; even hydrogels slow to set are usable because the material is constrained by the mold’s geometry. Common hydrogels, and blends thereof, used for injection molding include collagen, gelatin, alginate, hyaluronic acid, PLGA and agarose [138,140,141]. While injection molding has been shown to be a cost-effective tool for rapidly fabricating 3D tissue constructs to a specific overall geometry, it offers little to no spatial control of cells or other suspended contents, nor the ability to create internal architecture. In order to better control spatial composition, layer-by-layer techniques have been utilized to fabricate 3D constructs. Such 3D constructs are created using crosslinkable hydrogels and conventional patterning techniques, performed in a layer-by-layer fashion. To perform layer-by-layer printing, inkjet and LDW users have developed different fabrication methods. Inkjet users have achieved layer-by-layer fabrication with continuous extrusion of hydrogel precursor strand into a crosslinking agent [142]. A different technique prints droplets of hydrogel precursor onto a receiving substrate, then crosslinks the layer using a nebulizer [111,143]. The crosslinked layer is then used as the new printing surface. In the case of LDW, researchers are approaching layer-by-layer fabrication by coating their substrates in a hydrogel precursor and/or partially crosslinking the hydrogel layer [131,144], patterning, then cross-linking the cell suspension, then adding an additional hydrogel layer. Similar to inkjet printing, the printed hydrogel layer serves as the new printing surface, thereby allowing thick, multiple-layered, structures to be achieved. Furthermore, the distance between cell layers can be controlled by using a blade coater to adjust the height of hydrogel precursor [12]. 

A new generation of biosensing devices could be realized by applying the various 3D biofabrication technologies to incorporate biosensors. If cell-based biosensors are utilized, 3D microenvironments can prove more physiologically relevant than 2D substrates. Multiplexing components of the biosensor, such as testing an analyte against multiple cell types in 3D configurations, or multiple analytes against one cell type, may yield more relevant results if cells are in a 3D microenvironment. This sort of multiplexing yields much more information than independently testing a single analyte with a single cell type in a single biomaterial. Only by 3D patterning is robust, *in vitro*, 3D, high-throughput, cell-based biosensing possible. There may be additional benefits to fabricating 3D biosensors. Analytes traveling through a bulk structure made of natural polymeric material will experience more physiologically-relevant diffusive properties than those in 2D, which can influence temporal aspects and the responsiveness of the biosensor. In situations where a biosensor is made to mimic natural tissue, this may prove to be an important feature to replicate. These structures have the additional potential to even be implanted for real-time sensing *in vivo*. Further, with layer-by-layer printing technology, various cell types inside the bulk material can be spatially arranged to produce a signal cascade, where one cell type acts as a transducer and gives feedback to a secondary cell type to perform a desired action. When faster analyte detection is desired, channels can be constructed to facilitate movement of the analyte to the transducer [95]. This capability could prove quite useful in a combined biosensor/therapeutic application. For example, a 3D cell-based sensor could be fabricated for diabetics, in which sugar levels could be detected rapidly by the insulin-producing beta-cells embedded in an implanted construct. These cells could trigger an insulin response to adjust blood sugar levels accordingly. With 3D fabrication technologies, it becomes possible to combine biosensors with a therapeutic treatment. 

## 5. Conclusions and Future Direction of the Field

As the demands and application of biosensing advance, the incorporation of biofabrication technologies into biosensor elements will be paramount. In order to multiplex a variety of signals and evaluate cellular responses in 2D and 3D, sophisticated transducers must be able to separate and quantify analytes of interest. There have been numerous recent advances to transducer technology in recent years. By combining these advances with the latest enabling biofabrication approaches, particularly contact-based and non-contact-based patterning, even further advances in biosensing technology can be achieved. This combination of sensing and fabrication advances can lead to the next generation of biosensors, with a greater degree of sensitivity, throughput, and dynamic range within a single sensor. Approaches such as microcontact printing, inkjet printing, or LDW enable specific analytes to be placed in distinct locations, thereby allowing the responses on a single cell type to be analyzed quickly. Similar approaches can be used with multiple cell types. Advances in non-contact printing can profoundly impact 3D cell-based biosensing, which may provide more physiologically relevant cellular responses. Layer-by-layer and microbead approaches will allow the fabrication of 3D biosensors, with all the advantages of multiplexing. As biosensors move to 3D, there is even the potential to incorporate biosensors into implantable therapeutics. The synergy of advances in biosensing and biofabrication, much more than the advances of these approaches individually, has the potential to be very powerful for future sensing, research, diagnostic, and therapeutic applications.
